# The Differential Effects of Repetitive Magnetic Stimulation in an *In Vitro* Neuronal Model of Ischemia/Reperfusion Injury

**DOI:** 10.3389/fneur.2018.00050

**Published:** 2018-02-13

**Authors:** Ahreum Baek, Ji Hyun Kim, Soonil Pyo, Joon-Ho Jung, Eun Jee Park, Sung Hoon Kim, Sung-Rae Cho

**Affiliations:** ^1^Department and Rehabilitation Medicine, Yonsei University Wonju College of Medicine, Wonju, South Korea; ^2^Department and Research Institute of Rehabilitation Medicine, Yonsei University College of Medicine, Seoul, South Korea; ^3^Brain Korea 21 PLUS Project for Medical Science, Yonsei University, Seoul, South Korea; ^4^Department of Medicine, Yonsei University College of Medicine, Seoul, South Korea; ^5^Department of Rehabilitation Medicine, Graduate School Yonsei University Wonju College of Medicine, Wonju, South Korea; ^6^Yonsei Stem Cell Center, Avison Biomedical Research Center, Seoul, South Korea; ^7^Rehabilitation Institute of Neuromuscular Disease, Yonsei University College of Medicine, Seoul, South Korea

**Keywords:** repetitive magnetic stimulation, *in vitro* neuronal model of ischemia/reperfusion injury, extracellular signal-regulated kinases and AKT signaling pathway, apoptosis, Ca^2+^–calmodulin-dependent protein kinase II-cAMP-response element binding protein signaling pathway, brain-derived neurotrophic factor, synaptic plasticity, high frequency

## Abstract

Repetitive transcranial magnetic stimulation (rTMS) is a non-invasive therapy that has been implicated in treatment of serious neurological disorders. However, the neurobiological mechanisms underlying the effects of rTMS remain unclear. Therefore, this study examined the differential effects of repetitive magnetic stimulation (rMS) in an *in vitro* neuronal model of ischemia/reperfusion (I/R) injury, depending on low and high frequency. Neuro-2a cells were differentiated with retinoic acid and established for *in vitro* neuronal model of I/R injury under a subsequent 3 h of oxygen and glucose deprivation/reoxygenation (OGD/R) condition. After the I/R injury, the differentiated neuronal cells were stimulated with rMS on day 1 and randomly divided into three groups: OGD/R+sham, OGD/R+low-frequency, and OGD/R+high-frequency groups. High-frequency rMS increases cell proliferation through activation of extracellular signal-regulated kinases and AKT-signaling pathway and inhibits apoptosis in OGD/R-injured cells. Furthermore, high-frequency rMS increases Ca^2+^–calmodulin-dependent protein kinase II (CaMKII)-cAMP-response element binding protein (CREB) signaling pathway, further leading to alternation of brain-derived neurotrophic factor expression and synaptic plasticity in OGD/R injured cells. These results verified the neurobiological mechanisms of frequency-dependent rMS in I/R injury-treated neuronal cells. These mechanisms will help develop more powerful and credible rTMS stimulation treatment protocols.

## Introduction

Magnetic stimulation produces current flow in the nerve tissue and causes neuronal depolarization (1, 2). Transcranial magnetic stimulation (TMS) generates current flow in the brain without direct contact with the scalp and can be used to assess and control the excitability of certain regions of the brain (1, 3). When induced at a regular frequency, these TMS pulses are called repetitive transcranial magnetic stimulation (rTMS) (4).

rTMS is a non-invasive and less painful method to induce brain stimulation with no significant side effects (1, 5). rTMS is used as a treatment for a wide range of neurologic diseases, such as stroke and movement disorders, psychiatric diseases, and pain syndromes (6).

Several studies have demonstrated that the excitability of the cortex can be differentially modulated by intensity, frequency, and the overall pattern of the rTMS (3). Frequency is an important factor that can control cortical excitability. High-frequency (>3 Hz) stimulation usually has an effect of facilitation while low-frequency (≤1 Hz) stimulation has a lowering effect of synaptic efficiency (7–11).

In stroke patients, the motor dysfunction of paretic limb is accompanied by decreased ipsilesional cortical excitability and increased interhemispheric inhibition (IHI) due to the increased contralesional cortical excitability (12). Therefore, rTMS in stroke patients can improve the function of paretic limb by increasing ipsilesional cortical excitability by applying high frequency rTMS to ipsilesional hemisphere (13–15). There are also several studies that improve the excitability of the ipsilesional cortex *via* the reduction of the IHI by applying low frequency rTMS to the contralesional hemisphere to improve the function of the paretic limb (16–20).

Furthermore, rTMS treatment is known to affect the regulation of brain plasticity in ischemic stroke patients (21). There are several studies to support neurotrophic factor-mediated brain plasticity as to a mechanism of stroke rehabilitation, and it is known that the expression of brain-derived growth factor (BDNF), which plays an important role in brain plasticity, changes in association with synaptic activity (22). In addition, several *in vitro* and *in vivo* studies have shown that rTMS affects the expression of various neurotrophic/growth factors, including BDNF, and neuroblastoma cell proliferation, which has been verified by the various frequencies of rTMS (23–25).

In ischemic stroke, brain injury is caused by ischemia as well as cell damage induced by reperfusion injury (26). Oxygen and glucose deprivation/reoxygenation (OGD/R) is well established in an *in vitro* model for the study of ischemic/reperfusion (I/R) injury of neurons (27, 28). A previous research confirms that the injury induced by OGD/R can mimic the I/R injury in an *in vivo* model of ischemic stroke (29).

Although considerable research has been done on the therapeutic use of rTMS for brain ischemic injury, the precise mechanism is still elusive. Therefore, to understand the therapeutic effect and mechanism of rTMS, it is necessary to combine the mechanism based on brain plasticity. In this study, we aimed to investigate the differential effects of repetitive magnetic stimulation (rMS) depending on frequency in an *in vitro* neuronal model of I/R injury using OGD/R.

## Materials and Methods

### Cell Cultures

Neuro-2a (N2a) cells were purchased from American Type Culture Collection biotechnology (ATCC, Manassas, VA, USA). N2a cells were derived from mouse neuroblastoma, which exhibits properties of neuronal stem cells and could differentiate into neuronal cells when treated with retinoic acid (RA). N2a cells were maintained in growth medium, which were Dulbecco’s Modified Eagle Medium (DMEM; Hyclone, Logan, UT, USA) containing 10% fetal bovine serum (FBS; Serum Source International, Charlotte, NC, USA) and 1% Penicillin–Streptomycin solution (Gibco, Rockville, MD, USA), in a humidified 5% CO_2_ atmosphere at 37°C. When N2a cells reached 70–80% confluency, the medium was changed into differentiation medium, which contain 2% FBS and 20 µM of RA in DMEM, for 4 days. Differentiated N2a cells were maintained in a humidified atmosphere of 5% CO_2_ at 37°C, and the differentiation medium was changed every 2 days.

### OGD/R and rMS

**Figure 1:**
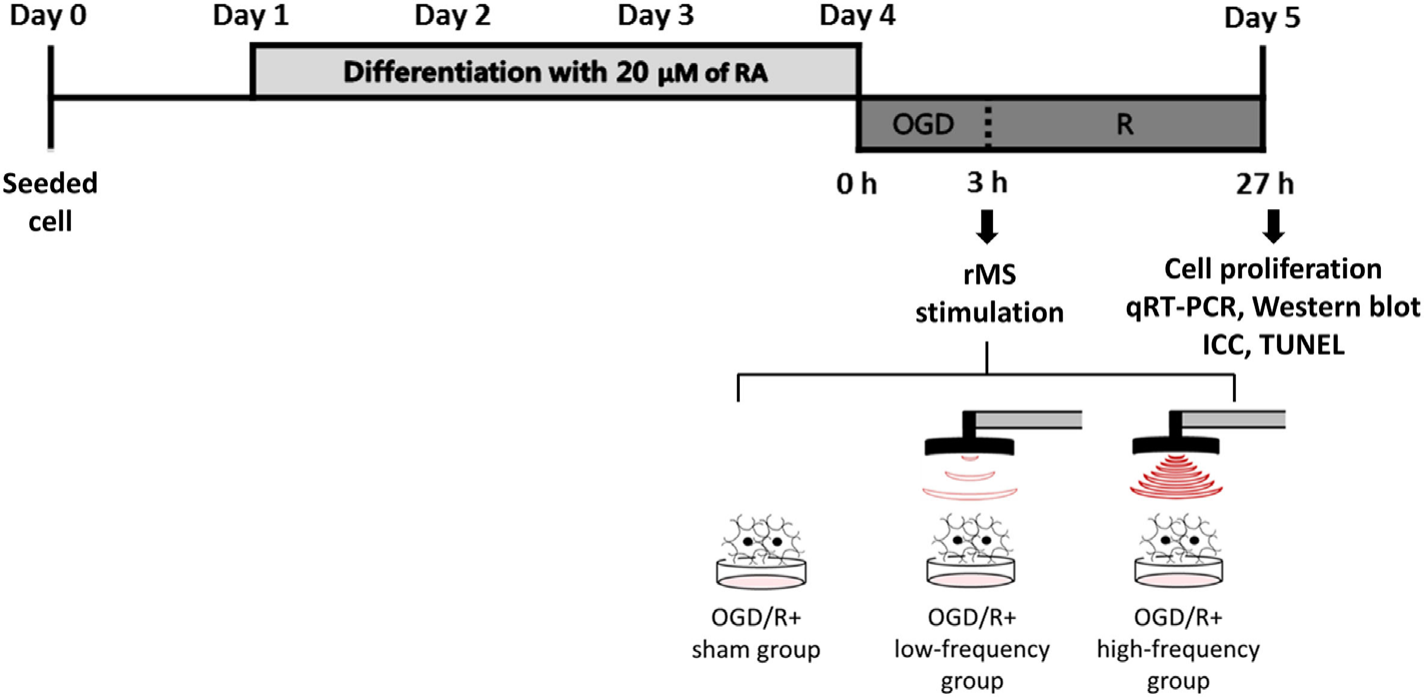
The experimental timeline of oxygen and glucose deprivation/reoxygenation (OGD/R) exposure and rMS stimulation. On day 0, the equal amount of Neuro-2a (N2a) cells were seeded in growth medium, which contains 10% fetal bovine serum (FBS) and 1% Penicillin–Streptomycin solution. At 70–80% confluency, the growth medium of N2a cells was changed into differentiation medium, which contains 20 µM of retinoic acid (RA) in Dulbecco’s Modified Eagle Medium (DMEM) for 4 days. After washing three times with PBS, the differentiation medium of N2a cells was changed into deoxygenated, glucose-free balanced salt solution (Gibco) and placed into a hypoxic chamber (O_2_ tension 1%) for 3 h. After this OGD injury, the cells were placed with medium and received on–off interval of 3 s stimulation for 10 min. After the stimulation, the cells were incubated in a humidified atmosphere of 5% CO_2_ at 37°C. After 24 h, OGD/R+rMS cells were harvested for cell proliferation analysis, qRT-PCR, Western blot, ICC, and TUNEL assay. RA, retinoic acid; OGD/R, oxygen and glucose deprivation/reperfusion; rMS, repetitive magnetic stimulation; qRT-PCR, quantitive real-time reverse transcription polymerase chain reaction; ICC, immunocytochemistry; TUNEL, terminal dUTP nick end-labeling.

**Figure 2:**
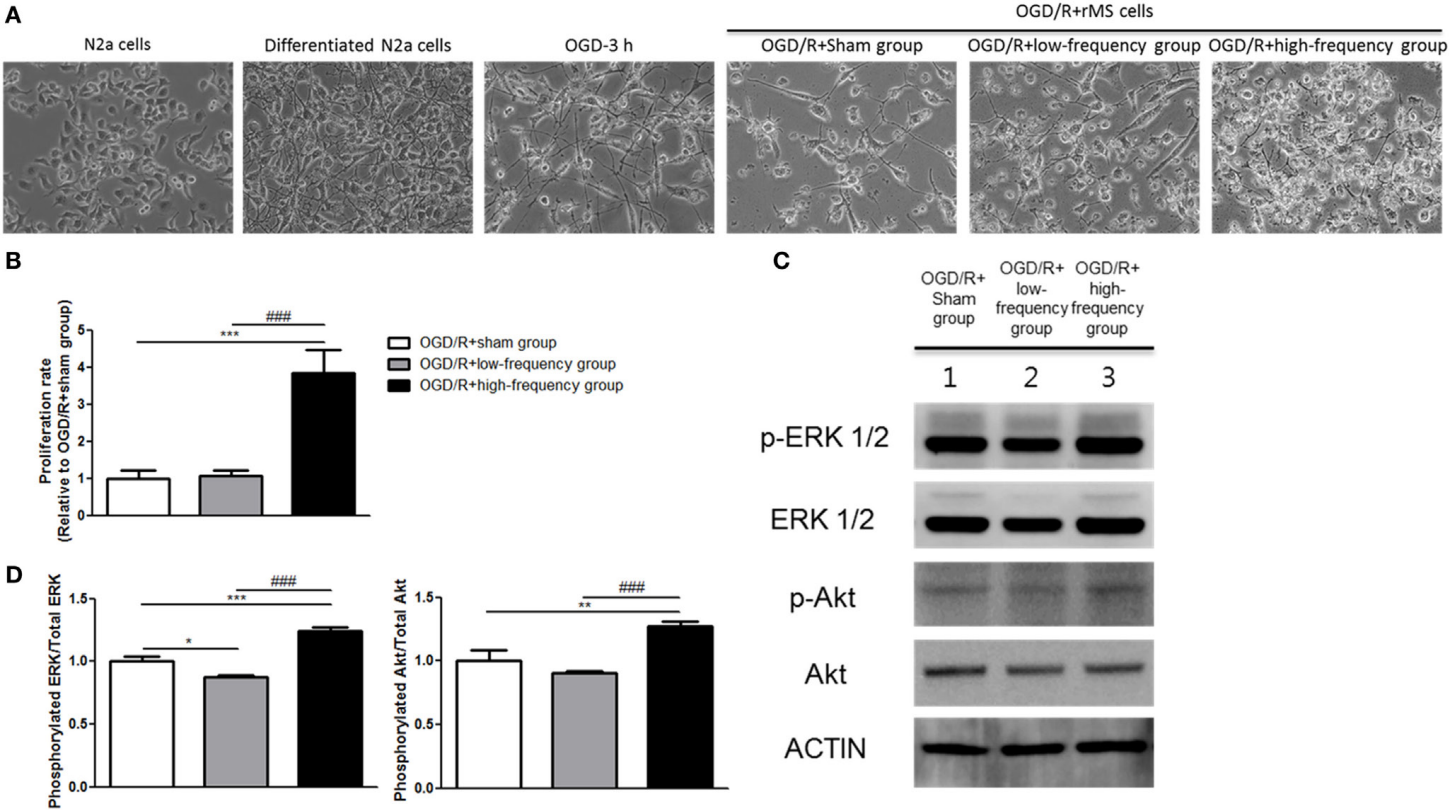
High-frequency repetitive magnetic stimulation (rMS) increases proliferation through the extracellular signal-regulated kinase (ERK) and AKT signaling pathway following oxygen and glucose deprivation/reoxygenation (OGD/R) injury. **(A)** Changed morphology of the cells was observed under microscope and photographed with a digital imaging system. **(B)** Bar graphs were shown for the proliferation rate of rMS stimulation following OGD/R injury. The white bars indicate OGD/R+sham group, the gray bars indicate OGD/R+low-frequency group, and the black bars indicate OGD/R+high-frequency group. Proliferation rate was represented as the mean ± SEM (OGD/R+sham group as control). ***p < 0.001 comparison with the OGD/R+sham group. ^###^*p* < 0.001 comparison with the OGD/R+low-frequency group. **(C)** Western blot analysis with antibodies against p-ERK, t-ERK, p-AKT, t-AKT, and ACTIN. **(D)** Comparison of relative expression for p-ERK/ERK and p-AKT/AKT from the OGD/R+rMS-treated group versus the OGD/R+sham group. All results are expressed as means ± SEM. *p < 0.05, **p < 0.01, and ***p < 0.001 comparison with the OGD/R+sham group. ^###^*p* < 0.001 comparison with the OGD/R+low-frequency group.

The following procedures have been adapted from previous studies (29–31). Confluent-differentiated N2a cells were washed three times with phosphate-buffered saline (PBS) and the differentiation medium was replaced with deoxygenated, glucose-free balanced salt solution (Gibco), and transferred to a hypoxic chamber (O_2_ tension 1%) for 3 h. Following OGD, injured cells were replaced with growth medium and stimulated with customized rMS (Bicon-1000Pro, Mcube Technology, Seoul, Korea). The magnetic coil (70-mm diameter) was placed and positioned 1 cm away from the cell culture dish. OGD/R cells were divided into three groups, as the OGD/R+sham group (placing the culture dishes without magnetic stimulation), the OGD/R+low-frequency group (0.5 Hz stimulation, on–off interval of 3 s), and the OGD/R+high-frequency group (10 Hz stimulation, on–off interval of 3 s) for 10 min. After the stimulation, cells were incubated in a humidified atmosphere of 5% CO_2_ at 37°C. After 24 h, OGD/R+rMS cells were harvested using 0.25% trypsin-EDTA (Gibco). The experimental schedule is presented in Figure 1. All procedures of the cells were observed under microscope and changed morphology was photographed with a digital imaging system (Eclipse TS100; Nikon USA, Melville, NY, USA) in Figure 2A.

### Cell Proliferation

To analyze the proliferation of the cells, the number of OGD/R+rMS cells was calculated with an advanced detection and accurate measurement (ADAM) automatic cell counter (NanoEnTek Inc., Seoul, South Korea).

### RNA Preparation

Total RNA was prepared in the whole cell lysates using TRIzol reagent (Invitrogen Life Technologies, Carlsbad, CA, USA) according to the manufacturer’s instructions. A nanodrop spectrophotometer (Thermo Fisher Scientific, Waltham, MA, USA) were used to confirm the quality and quantity of extracted RNA.

### Quantitative Real-Time Reverse Transcription Polymerase Chain Reaction (qRT-PCR)

To examine mRNA expression, qRT-PCR was conducted. ReverTra Ace^®^ qPCR RT Master Mix with gDNA Remover (Toyobo, Osaka, Japan) was used to synthesize cDNA with total RNA. The procedures were according to the manufacturer’s instructions. qPCR BIO SyGreen Mix Hi-ROX (PCR BIOSYSTEMS, London, UK) was used to confirm the mRNA expression for genes of interest in a StepOnePlus Real-Time PCR System (Applied Biosystems, Foster City, CA, USA). Data analysis was performed using the 2^−ΔΔCT^ method (32). Primers used for qRT-PCR are described in Table 1.

**Table 1:** Primers used for quantitative real-time reverse transcription polymerase chain reaction (qRT-PCR).

Gene symbol	Forward primer (5′ → 3′)	Reverse primer (5′ → 3′)
*GRIN1*	CAG GAT CGT CAG GCA AGA CA	CCA AGC AAC TGA GGG TCC TT
*CaMKIIα*	TGC TGC TCT TTC TCA CGC TG	TCA ATG GTG GTG TTG GTG CT
*CaMKII*δ	TGC ACC TGG TAG GGG ACG AT	GAA TAC AGG GTG GCT TGA TGG GT
*BDNF*	GGG TCA CAG CGG CAG ATA AA	ATT GCG AGT TCC AGT GCC TT
*Synaptophysin*	GTG CCA ACA AGA CGG AGA GT	CAC CCG AGG AGG AGT AGT CA
*PSD-95*	TCC CCA TTT TCT CCC ACA CAC	ACG GCG TGG GGA GTT ATG AT
*GAPDH*	CAT CAC TGC CAC CCA GAA GAC TG	ATG CCA GTG AGC TTC CCG TTC AG

*Grin 1, glutamate receptor ionotropic N-methyl-d-aspartate 1; CaMKIIα, calcium/calmodulin-dependent protein kinase II alpha; CaMKIIδ, calcium/calmodulin-dependent protein kinase type II subunit delta; BDNF, brain-derived neurotrophic factor; PSD-95, postsynaptic density protein-95; GAPDH, glyceraldehyde-3-phosphate dehydrogenase*.

### Western Blot

To confirm the protein expression, whole cell lysates were homogenized and dissolved with radioimmunoprecipitation assay buffer (Thermo Scientific) containing protease and phosphatase inhibitors (Abcam, Cambridge, MA, USA). The BCA™ Protein Assay Kit (Thermo Scientific) was used to quantify the total protein. The samples were denatured and separated by 4–12% Bis-Tris gels in 1× NuPage MES SDS Running Buffer (Invitrogen, Eugene, OR, USA). Proteins were transferred at 4°C onto a polyvinylidene difluoride membrane (Invitrogen) in NuPage Transfer Buffer (Invitrogen) with 20% (vol/vol) methanol. Membranes were blocked and then incubated overnight at 4°C with the following antibodies: Anti-p-Erk1/2 (1:1,000 dilution, Santa Cruz Biotechnology, Santa Cruz, CA, USA), Anti-t-Erk1/2 (1:1,000 dilution, Santa Cruz Biotechnology), Anti-p-AKT (1:1,000 dilution, Santa Cruz Biotechnology), Anti-t-AKT (1:1,000 dilution, Santa Cruz Biotechnology), Anti-BAX (1: 1,000 dilution, Santa Cruz Biotechnology), Anti-Bcl-2 (1:1,000 dilution, Santa Cruz Biotechnology), Anti-Cleaved-caspase-3 (1:1,000 dilution, Cell Signaling Technology, Danvers, MA, USA), Anti-Pro-caspase-3 (1:1,000 dilution, Santa Cruz Biotechnology), Anti-*N*-methyl-d-aspartate receptors1 (NMDAR1; 1: 1,000 dilution, Invitrogen), Anti-CaMKII (1:1,000 dilution, Abcam), Anti-CREB (1:1,000 dilution, Santa Cruz Biotechnology), Anti-p-CREB (1:1,000 dilution, Santa Cruz Biotechnology), Anti-BDNF (1:1,000 dilution, Abcam), and Anti-ACTIN (1:5,000 dilution, Santa Cruz Biotechnology). The next day, blots were washed three times with TBS plus Tween 20 and incubated at room temperature for 1 h with horseradish peroxidase-conjugated secondary antibodies (1:4,000 dilution, Santa Cruz). An enhanced chemiluminescence detection system (Amersham Pharmacia Biotech, Little Chalfont, UK) was used to visualize the protein.

### Immunocytochemistry (ICC)

To confirm the expression of BDNF, Synaptophysin and postsynaptic density protein-95 (PSD-95) in differentiated N2a cells were seeded into 0.2% gelatin (Sigma-aldrich, St. Louis, MO, USA) in PBS coated 4-well with OGD/R+rMS stimulation as described above. Cells were stained with Anti-BDNF (1:400 dilution, Abcam), Anti-Synaptophysin (1:400 dilution, Abcam), and Anti-PSD-95 (1:400 dilution, Abcam) and incubated with Alexa Fluor^®^ (1:400, Invitrogen) secondary antibody. Samples were mounted on glass slides with fluorescent mounting medium with 4′,6-diamidino-2-phenylindole (DAPI; Vectorshield, Vector Laboratories, Burlingame, CA, USA) for imaging using the Zeiss Axio Imager M2 (Carl Zeiss, Gottingen, Germany) fluorescence microscope.

### Terminal dUTP Nick End-Labeling (TUNEL) Assay

For analysis of apoptosis, differentiated N2a cells were seeded into gelatin (Sigma-aldrich) coated 4-well. OGD/R and rMS stimulation were performed as described above. Colorimetric TUNEL assay (Promega, Madison, WI, USA) was conducted according to the manufacturer’s protocol.

### Statistical Analysis

All experiments were conducted at least three independent replications. The results were expressed as means ± SEM. The Statistical Package for Social Sciences version 23.0 was used for statistical analysis. The one-way analysis of variance, followed by Bonferroni *post hoc* multi-comparison test was carried out to confirm the statistical results. Statistical differences were considered significant when *p* < 0.05.

## Results

### High-Frequency rMS Increases Cell Proliferation through the Activation of Extracellular Signal-Regulated Kinase (ERK) and AKT Signaling Pathway after OGD/R Injury

In the previous study (24), rMS affects cell proliferation in the neuroblastoma cells. Therefore, we evaluated cell proliferation with an ADAM, the automatic cell counting machine, after the stimulation with or without rMS following OGD/R injury. After 24 h following OGD/R+rMS stimulation, the proliferation rate of the OGD/R+low-frequency group (1.06 ± 0.16) was not significantly different compared to the OGD/R+sham group (Figure 2B). However, in the OGD/R+high-frequency group, proliferation rate was significantly increased compared to the OGD/R+sham group, and the proliferation rate and statistical value were as follows: 3.84 ± 0.64, *p* < 0.001 (Figure 2B). In the same manner, the proliferation rate of the OGD/R+high-frequency group was significantly increased compared to the OGD/R+low-frequency group (*p* < 0.001) (Figure 2B).

Next, we investigated ERK and AKT pathway, which were known to play an important role in the development of synaptic plasticity and neurotrophic activity (33) as well as the regulation of growth and proliferation (24, 34), by western blot analysis (Figure 2C). The OGD/R+low-frequency group showed the decreased expression of p-ERK and p-AKT compared to the OGD/R+sham group and the expression values were as follows: p-ERK/ERK (0.88 ± 0.01, *p* < 0.05), p-AKT/AKT (0.90 ± 0.01, *p* < 0.01) (Figure 2D). By contrast, the OGD/R+high-frequency group showed the significantly activated p-ERK and p-AKT expression compared to the OGD/R+sham group, and the expression values were as follows: p-ERK/ERK (1.25 ± 0.02, *p* < 0.001) and p-AKT/AKT (1.27 ± 0.04, *p* < 0.01) (Figure 2D). The OGD/R+high-frequency group also showed the significant increased expression of p-ERK and p-AKT compared to the OGD/R+low-frequency group and the expression values were as follows: p-ERK/ERK (*p* < 0.001), p-AKT/AKT (*p* < 0.001) (Figure 2D).

Thus, we suggest that high frequency rMS increases cell proliferation *via* activation of ERK and AKT signaling pathway following OGD/R injury in differentiated neuronal cells.

### High-Frequency rMS Reduces Apoptotic Process after OGD/R Injury

**Figure 3:**
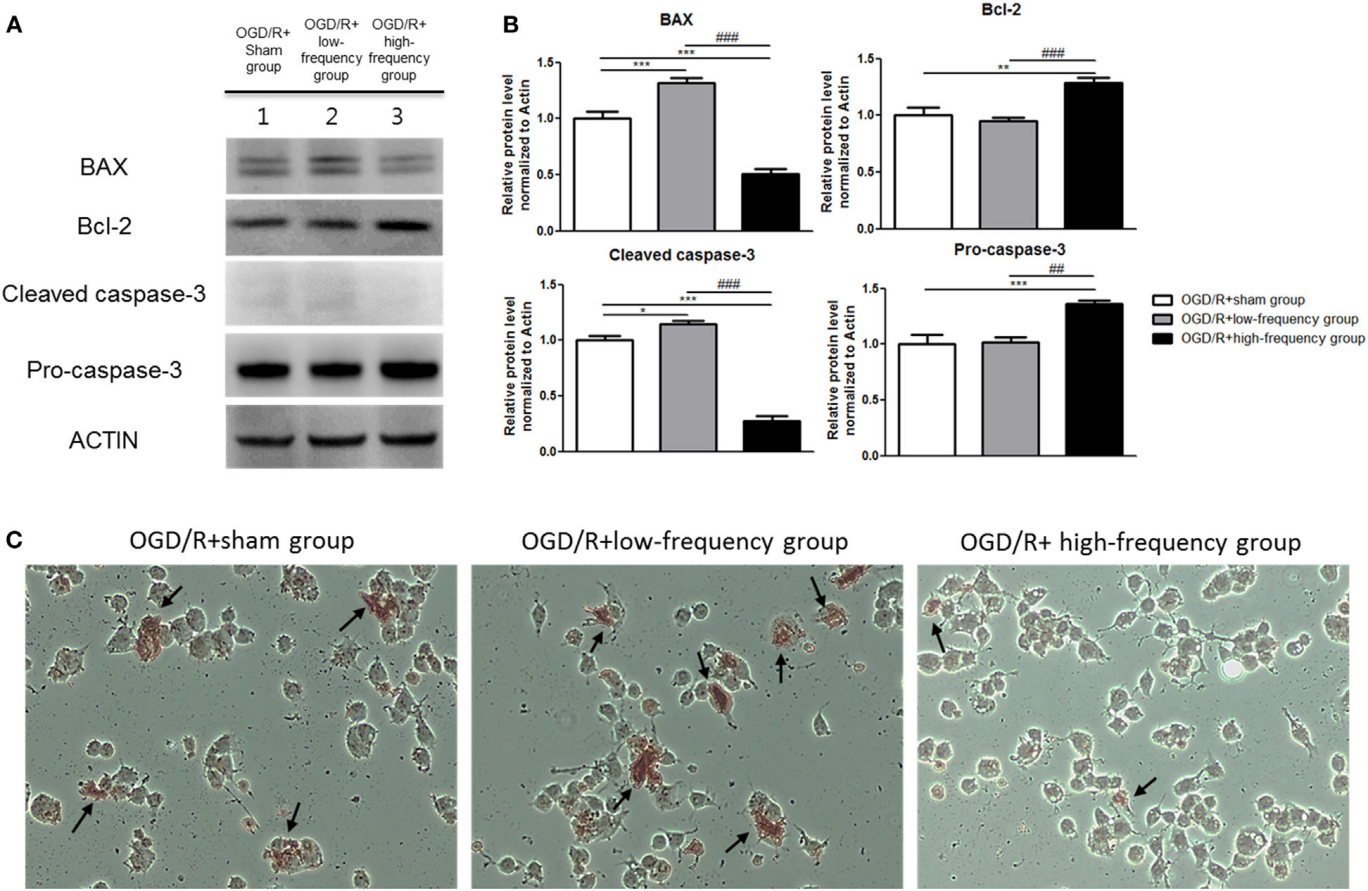
High-frequency repetitive magnetic stimulation (rMS) inhibits apoptosis expression following oxygen and glucose deprivation/reoxygenation (OGD/R) injury. **(A)** Western blot analysis with antibodies against Bcl-2-associated X protein (BAX), Bcl-2, Cleaved caspase-3, and Pro-caspase-3. **(B)** Comparison of relative expression apoptosis protein from the OGD/R+rMS treated group versus the OGD/R+sham group. All results are expressed as means ± SEM. *p < 0.05, **p < 0.01 and ***p < 0.001 comparison with the OGD/R+sham group. ^##^*p* < 0.01 and ^###^*p* < 0.001 comparison with the OGD/R+low-frequency group. **(C)** Detection of apoptosis with terminal dUTP nick end-labeling (TUNEL) assay in the rMS stimulation following OGD/R injury. Black arrow indicates apoptotic nuclei following OGD/R injury in differentiated Neuro-2a (N2a) cells.

It has been well demonstrated that neuronal apoptosis is one of the hallmarks of cerebral ischemia/reperfusion (I/R) injury (35). We constructed the OGD/R injury in differentiated neuronal cells to mimic the *in vivo* cerebral I/R conditions. Apoptotic process was investigated using the protein expressions of apoptosis-related proteins, such as Bcl-2-associated X protein (BAX), Bcl-2, Cleaved caspase-3, and Pro-caspase-3 (Figure 3A). As shown in Figures 3A,B, the OGD/R+high-frequency group showed significant decrease in the expression of pro-apoptotic proteins, such as BAX and Cleaved-caspase-3, while showing significant increase in the expression of anti-apoptotic proteins, as Bcl-2 and Pro-caspase-3, compared to the OGD/R+sham group. The expression values were as follows: BAX (0.50 ± 0.08, *p* < 0.001), Bcl-2 (1.29 ± 0.04, *p* < 0.01), Cleaved-caspase-3 (0.28 ± 0.04, *p* < 0.001), and Pro-caspase-3 (1.36 ± 0.03, *p* < 0.001). In the OGD/R+low-frequency group compared to the OGD/R+sham group, the expression of pro-apoptotic proteins, such as BAX and Cleaved-caspase-3, was increased, while the expression of anti-apoptotic proteins, such as Bcl-2 and Pro-caspase-3, was decreased. The expression values were as follows: BAX (1.32 ± 0.04, *p* < 0.001), Bcl-2 (0.95 ± 0.03), Cleaved-caspase-3 (1.15 ± 0.02, *p* < 0.05), and Pro-caspase-3 (1.02 ± 0.05). In the same manner, in the OGD/R+high-frequency group compared to the OGD/R+low-frequency group, the expression of pro-apoptotic proteins was statistically decreased, while the expression of anti-apoptotic proteins was statistically increased. The expression statistical values were as follows; BAX (*p* < 0.001), Bcl-2 (*p* < 0.001), Cleaved-caspase-3 (*p* < 0.001), and Pro-caspase-3 (*p* < 0.01).

To further investigate the underlying mechanism associated with apoptosis, we used TUNEL assay (Figure 3C). The number of TUNEL-positive nuclei was much observed in the OGD/R+low-frequency group compared to the OGD/R+sham group or the OGD/R+high-frequency group. On the other hand, there were few TUNEL-positive nuclei in the OGD/R+high-frequency group compared to the OGD/R+sham group and the OGD/R+low-frequency group.

These data suggest that high frequency rMS inhibits the progression of apoptosis following OGD/R injury in differentiated neuronal cells.

### High-Frequency rMS Increases Ca^2+^–CaMKII–CREB Signaling Pathway after OGD/R Injury

**Figure 4:**
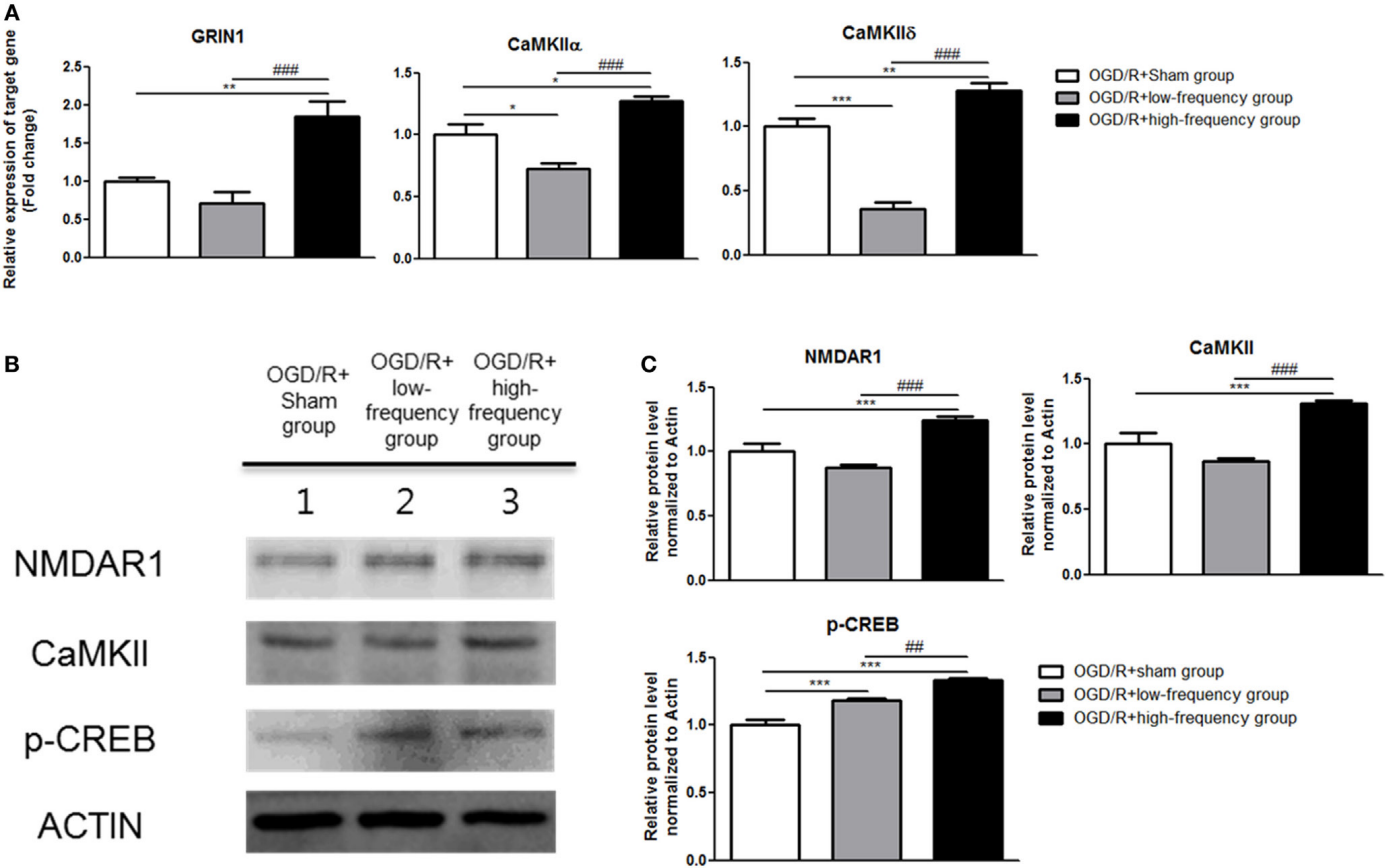
High-frequency repetitive magnetic stimulation (rMS) activates Ca^2+^–calmodulin-dependent protein kinase II (CaMKII)–cAMP-response element binding protein (CREB) signaling pathway following oxygen and glucose deprivation/reoxygenation (OGD/R) injury. **(A)** GRIN1, CaMKIIα, and CaMKIIδ expressions were examined by quantitative real-time reverse transcription polymerase chain reaction. The relative expression of target genes was normalized by the OGD/R+sham group and was calculated using 2^−ΔΔCt^ method. **(B)** Western blot analysis was performed using antibodies against *N*-methyl-d-aspartate receptors1(NMDAR1), CaMKII, p-CREB, and ACTIN (as control). **(C)** Comparison of relative expression apoptosis protein from the OGD/R+rMS-treated group versus the OGD/R+sham group. All results are expressed as means ± SEM. *p < 0.05, **p < 0.01, and ***p < 0.001 comparison with the OGD/R+sham group. ^#^*p* < 0.05, ^##^*p* < 0.01, and ^###^*p* < 0.001 comparison with the OGD/R+low-frequency group.

In our previous study, we confirmed with transcriptome analysis that high frequency rMS can activate Ca^2+^–CaMKII–CREB signaling pathway, thus increasing the expression of p-CREB and BDNF *via* the activation of that signaling pathway in the neuronal cells. We further evaluated Ca^2+^–CaMKII–CREB signaling pathway following OGD/R+rMS stimulation. By qRT-PCR, in the OGD/R+low-frequency group compared to the OGD/R+sham group, the expression of GRIN1, CaMKIIα, and CaMKIIδ was decreased (Figure 4A). The expression values as follows; GRIN1 (0.71 ± 0.15), CaMKIIα (0.72 ± 0.05, *p* < 0.05), and CaMKIIδ (0.36 ± 0.05, *p* < 0.001). In the OGD/R+high-frequency group, the expression of GRIN1, CaMKIIα, and CaMKIIδ was significantly increased compared to the OGD/R+sham group (Figure 4A). The expression values were as follows: GRIN1 (1.85 ± 0.20, *p* < 0.01), CaMKIIα (1.27 ± 0.04, *p* < 0.05), and CaMKIIδ (1.28 ± 0.06, *p* < 0.01). Furthermore, in the OGD/R+high-frequency group compared to the OGD/low-frequency group, the expression of GRIN1, CaMKIIα, and CaMKIIδ was significantly increased (Figure 4A). The statistical values were as follows: GRIN1 (*p* < 0.001), CaMKIIα (*p* < 0.001), and CaMKIIδ (*p* < 0.001).

Next, to further evaluate Ca^2+^–CaMKII–CREB signaling pathway, western blot was conducted (Figure 4B). In the OGD/R+low-frequency group, the expression of *N*-methyl-d-aspartate receptors1 (NMDAR1), CaMKII, and p-CREB was decreased or no difference was shown compared to the OGD/R+sham group (Figure 4C). The expression values as follows: NMDAR1 (0.87 ± 0.20), CaMKII (0.86 ± 0.02), and p-CREB (1.19 ± 0.02, *p* < 0.001). In the OGD/R+high-frequency group, the expression of NMDAR1, CaMKII, and p-CREB was increased compared to the OGD/R+sham group (Figure 4C). The expression values as follows: NMDAR1 (1.25 ± 0.03, *p* < 0.001), CaMKII (1.31 ± 0.02, *p* < 0.001), and p-CREB (1.33 ± 0.01, *p* < 0.001). In the OGD/R+high-frequency group, the expression of NMDAR1, CaMKII, and p-CREB was increased compared to the OGD/R+low-frequency group (Figure 4C). The statistical values were as follows: NMDAR1 (*p* < 0.001), CaMKII (*p* < 0.001), and p-CREB (*p* < 0.01).

Taken together, these data suggest that high frequency of rMS activates Ca^2+^–CaMKII–CREB signaling pathway following OGD/R injury in differentiated neuronal cells.

### High-Frequency rMS Increases BDNF Expression after OGD/R Injury

**Figure 5:**
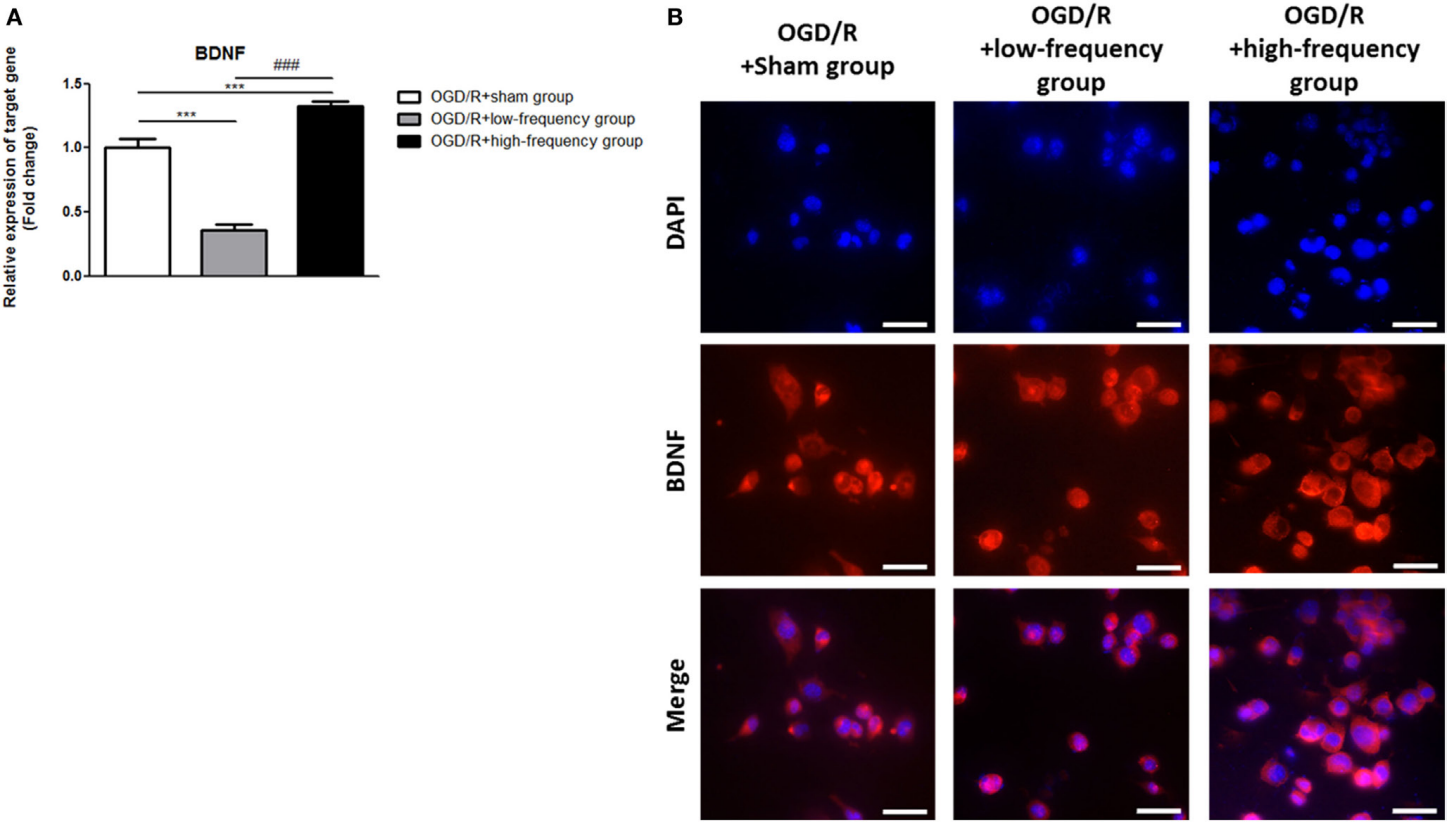
High-frequency repetitive magnetic stimulation increase brain-derived neurotrophic factor (BDNF) expression following oxygen and glucose deprivation/reoxygenation (OGD/R) injury. **(A)** BDNF mRNA expression was examined by quantitative real-time reverse transcription polymerase chain reaction (qRT-PCR). The relative expression of target gene was normalized by the OGD/R+sham group and was calculated using 2^−ΔΔCt^ method. **(B)** Immunocytochemistry (ICC) was conducted to evaluate the level of BDNF. Scale bar: 50 µM, BDNF: Red, 4′,6-diamidino-2-phenylindole (DAPI): Blue.

In the same manner, we examined the expression of BDNF following OGD/R+rMS stimulation. By qRT-PCR, we found that in the OGD/R+low-frequency group, the expression of BDNF was significantly decreased compared to the OGD/R+sham group, and the expression value was as a follow: BDNF (0.36 ± 0.05, *p* < 0.001) (Figure 5A). In the OGD/R+high-frequency group, the expression of BDNF was significantly increased compared to the OGD/R+sham group and the expression value was as a follow: BDNF (1.33 ± 0.04, *p* < 0.001) (Figure 5A). Furthermore, in the OGD/R+high-frequency group compared to the OGD/R+low-frequency group, the expression of BDNF was significantly increased and the statistical value was as follow: BDNF (*p* < 0.001) (Figure 5A).

Then, we further evaluated BDNF expression with ICC analysis (Figure 5B). In the OGD/R+high-frequency group, the protein level of BDNF is significantly higher than the OGD/R+low-frequency or sham group. BDNF expression was significantly decreased, whereas high frequency rMS induces BDNF expression following OGD/R injury in differentiated neuronal cells.

We conclude that high frequency rMS increases BDNF expression *via* activation of Ca^2+^–CaMKII–CREB signaling pathway following OGD/R injury in differentiated neuronal cells.

### High-Frequency rMS Increases Synaptic Plasticity after OGD/R Injury

**Figure 6:**
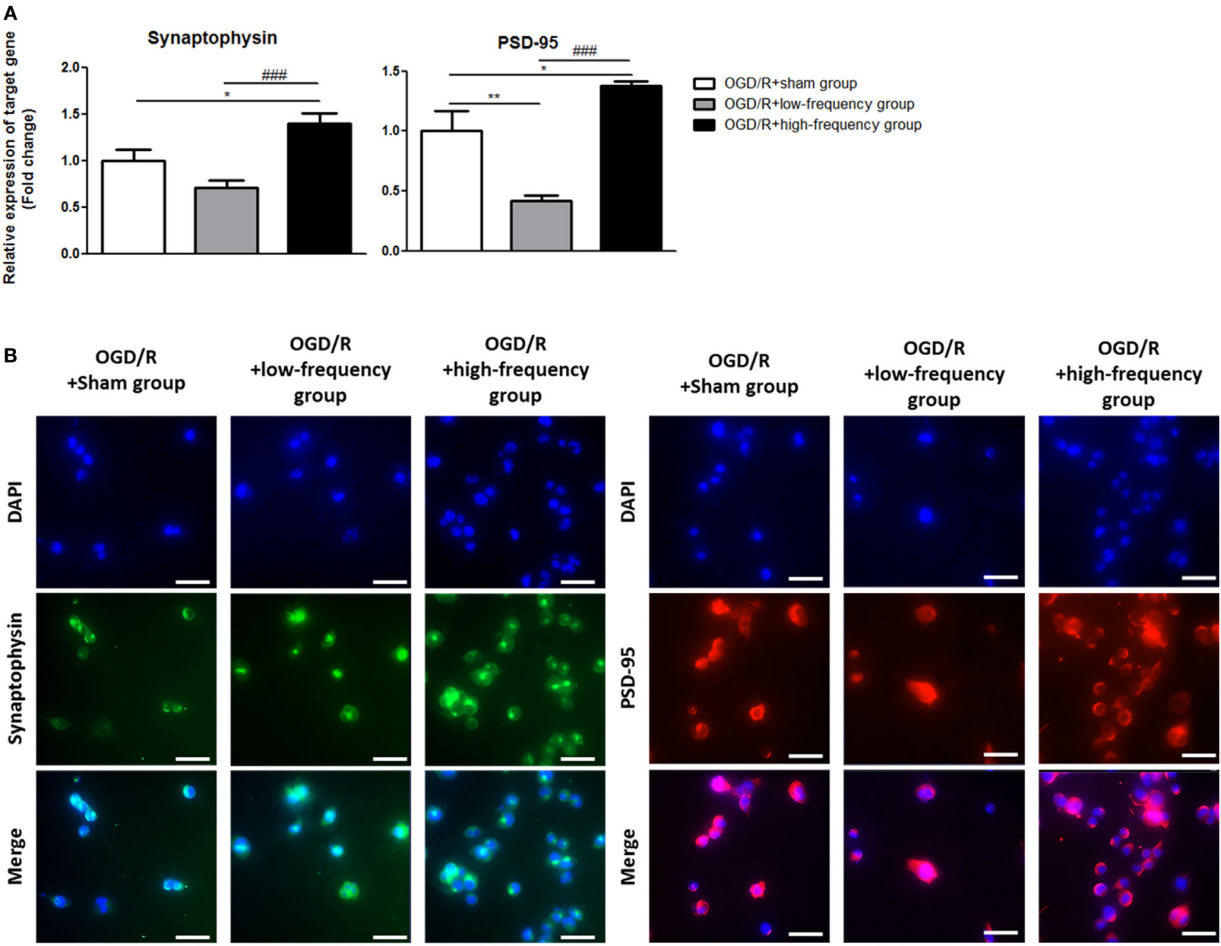
High-frequency repetitive magnetic stimulation increases synaptic plasticity following oxygen and glucose deprivation/reoxygenation (OGD/R) injury. **(A)** Synaptophysin and postsynaptic density protein-95 (PSD-95) expressions were examined by qRT-PCR. The relative expression of target genes was normalized with respect to the OGD/R+sham group and was calculated using 2^−ΔΔCt^ method. **(B)** Immunocytochemistry (ICC) was conducted to evaluate the level of synaptophysin and PSD-95. Scale bar: 50 µM, synaptophysin: Green, PSD-95: Red, 4′,6-diamidino-2-phenylindole (DAPI): Blue.

It is reported that the expression of p-CREB and BDNF is related to the regulation of synaptic plasticity (36, 37). We next assessed synaptic plasticity following OGD/R+rMS stimulation. By qRT-PCR, in the OGD/R+low-frequency group, the expression of synaptophysin, as a presynaptic marker, and PSD-95, as a postsynaptic marker, was significantly decreased compared to the OGD/R+sham group and the expression values were as follows: Synaptophysin (0.71 ± 0.07) and PSD-95 (0.41 ± 0.05, *p* < 0.01) (Figure 6A). In the OGD/R+high-frequency group, the expression of synaptophysin and PSD-95 was significantly increased compared to the OGD/R+sham group and the expression values were as follows: Synaptophysin (1.39 ± 0.12, *p* < 0.05) and PSD-95 (1.38 ± 0.04, *p* < 0.05) (Figure 6A). In the OGD/R+high-frequency group compared to the OGD/R+low-frequency group, the expression of synaptophysin and PSD-95 was significantly increased and the statistical values were as follows: Synaptophysin (*p* < 0.001) and PSD-95 (*p* < 0.001) (Figure 6A).

Next, we performed ICC analysis to evaluate synaptic plasticity (Figure 6B). In the OGD/R+low-frequency group, the protein level of synaptophysin and PSD-95 was not significantly different compared to the OGD/R+sham group. On the other hand, the protein level of synaptophysin and PSD-95 was significantly increased in the OGD/R+high-frequency group compared to the OGD/R+low-frequency or sham group.

These data indicated that high frequency rMS increases synaptic plasticity following OGD/R injury in differentiated neuronal cells.

## Discussion

Several studies have been conducted to study the effect of rMS on neuronal recovery in the I/R injury model (3, 25, 38, 39). However, the neurobiological mechanism of rMS on the frequency difference in the I/R injury model has not been fully understood. Frequency-dependent stimulation with the same duration has been reported as the primary treatment regime of rMS (38–43). In this study, we developed an I/R injury model using neuronal cells and then applied different frequencies of rMS to it.

It was found that in the high frequency of rMS, cell proliferation was increased, and apoptotic process was decreased compared to the sham or low-frequency rMS following I/R injury. We also verified that the activation of the Ca^2+^–CaMKII–CREB signaling pathway resulted in increased synaptic plasticity *via* increasing the expression of BDNF. These results are consistent with the previous studies using neuroblastoma cells, which were transformed neuron-liked cells (23, 24).

In this study, we constructed *in vitro* I/R injury model to verify accurate therapeutic mechanisms of rMS. When high-frequency rMS was applied to neuronal cells model with I/R injury, activation of the ERK and AKT signaling pathway promoted cell proliferation. These results are consistent with studies using neuroblastoma cells; the effects of rMS-induced cell proliferation were mediated by various growth factors, proliferation-, and survival-associated ERK and AKT (24). ERK and AKT signaling has been known to play an important role in the proliferation and maturation of neural progenitor cells (43, 44) as well as the regulation of migration of neuroblasts (45).

Next, we verified that high frequency of rMS inhibits the progression of apoptosis in differentiated neuronal cells model with I/R injury. This is in agreement with other *in vivo* studies. In a study of high-frequency rTMS using a subacute cerebral ischemic rat model, it was suggested that the anti-apoptotic mechanism is the main mechanism of rTMS treatment (39). Another study of the hippocampus area in an ischemic stroke rat model showed that high-frequency rTMS inhibited neuronal apoptosis in ischemic hemispheres (41). In this study, we verified *in vitro* that high-frequency rMS inhibits the progression of apoptosis, indicating a protective role for the neuronal cells, which have been subjected to I/R injury.

In the brain, BDNF is known to improve neuronal survival, synaptogenesis, angiogenesis, and neurogenesis to regulate neuroplasticity (22, 42, 46, 47). It is also known that rMS affects the expression of BDNF in cultured hippocampal neurons (23). In particular, high frequency of rMS has been shown to increase BDNF expression (24, 25). Our previous study has also shown that the increased expression of BDNF by high-frequency rMS is due to the activation of the Ca^2+^–CaMKII–CREB signaling pathway.

In this study, using the same method as our previous study, we verified that BDNF expression was increased by the activation of the Ca^2+^–CaMKII–CREB signaling pathway with high-frequency stimulation in the I/R injury model as well. We also examined that synaptophysin, a presynaptic marker, and PSD-95, a postsynaptic maker, were both found to increase in high-frequency rMS, thereby improving BDNF-mediated synaptic plasticity. It is thought that, when high-frequency rMS is applied, the various effects due to the increase expression of BDNF may act as a restoring mechanism of the function after I/R injury.

In this study, low-frequency rMS to neuronal cells with I/R injury showed no direct positive effects in cell proliferation, anti-apoptosis, and synaptic plasticity. Previous studies in stroke patients suggested that low-frequency rTMS stimulates the contralesional hemisphere rather than the lesion side, exerting the positive indirect effect and lowering hyper-excitability (48). Therefore, additional follow-up studies will be needed to investigate the molecular mechanism of low frequency rTMS in stroke.

These findings call for further studies on varying degrees of cell regeneration after rMS application, by comparing groups applied with low- and high-frequency rMS after I/R injury and groups neither applied with rMS nor subjected to I/R injury. It is necessary to conduct basic studies to evaluate the functional aspect of cells regenerated by high frequency rMS. In addition, there is a study indicating that the expression of genes involved in cell recovery, such as cell repair and structural remodeling, increases after rTMS in a rat model of cerebral I/R injury (3). Based on this, further studies on cell recovery mechanism after rMS application may be helpful to understand the mechanism of rTMS.

As to the limitation of this study, our data are restricted to the effect of low- and high-frequency rMS over the same duration, which has been investigated in many previous studies. The different parameters such as the number of magnetic stimuli and duration cannot be disregarded (49–51). Therefore, further prospective studies to evaluate the effect of rMS based on the different durations for both low- and high-frequency rMS are needed.

In this study, we verified the neurobiological mechanism of rMS on I/R injury in neuronal cells, which depends on frequency. These results are valuable as a basic study for understanding the mechanism of rTMS treatment for stroke patients. This mechanism may be useful in developing more robust and reliable rTMS treatment protocols.

## Conclusion

**Figure 7:**
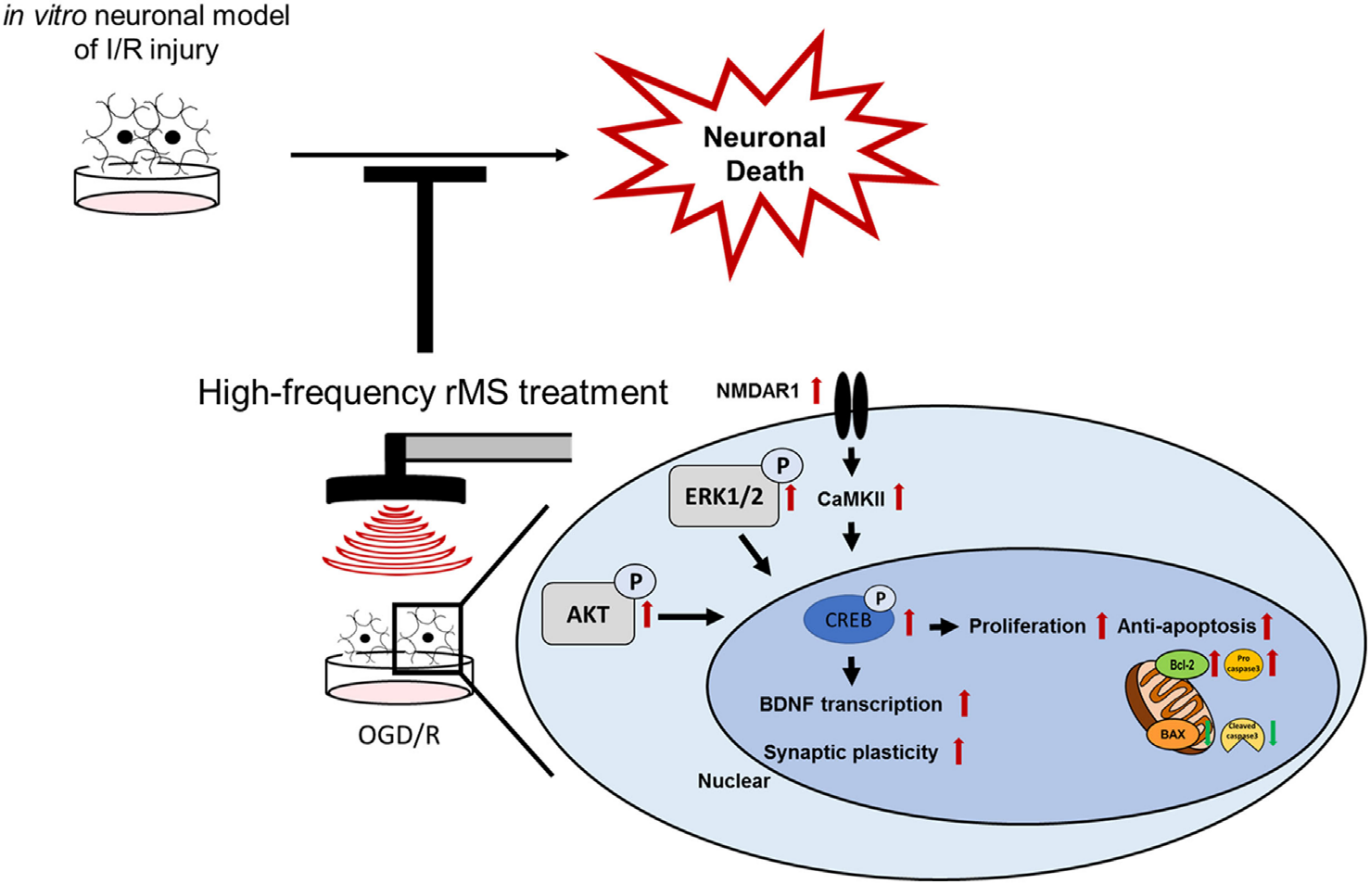
Schematic diagram of the therapeutic mechanisms of repetitive magnetic stimulation (rMS) following ischemia/reperfusion (I/R) injury. High-frequency rMS increases cell proliferation and mediates the protective effects against apoptosis induced by I/R injury. In addition to enhancing the Ca^2+^–calmodulin-dependent protein kinase II (CaMKII)–cAMP-response element binding protein (CREB) signaling pathway by high-frequency rMS, it also results in activation of brain-derived neurotrophic factor (BDNF) expression and synaptic plasticity in I/R injury.

Following I/R injury, neuronal cell death can be induced in an *in vitro* neuronal model (Figure 7). When high-frequency rMS is treated in the *in vitro* neuronal model of I/R injury, it can reduce neuronal cell death by increasing cell proliferation and anti-apoptosis. Furthermore, it can also increase BDNF expression and synaptic plasticity by activating the Ca^2+^–CaMKII–CREB pathway. These results demonstrated the combined mechanism of high-frequency rMS in the *in vitro* neuronal model, and its mechanism could also be applied to other neurological diseases.

## Author Contributions

AB contributed to study conception and design, collection and/or assembly of data, and manuscript writing. JHK contributed to manuscript writing and interpreted the data. SP contributed to manuscript writing and English editing. J-HJ and EJP contributed to collect and/or assembly of data. SHK and S-RC contributed to data analysis and interpretation, manuscript writing, and project supervision. All authors read and approved the manuscript.

## Conflict of Interest Statement

The authors declare that the research was conducted in the absence of any commercial or financial relationships that could be construed as a potential conflict of interest.
